# Extracellular HSPs: The Complicated Roles of Extracellular HSPs in Immunity

**DOI:** 10.3389/fimmu.2016.00159

**Published:** 2016-04-25

**Authors:** Stuart K. Calderwood, Jianlin Gong, Ayesha Murshid

**Affiliations:** ^1^Department of Radiation Oncology, Beth Israel Deaconess Medical Center, Harvard Medical School, Boston, MA, USA; ^2^Department of Medicine, Boston University Medical Center, Boston, MA, USA

**Keywords:** heat, shock, protein, immunity, immunosuppression, surface, receptors, scavenger

## Abstract

Extracellular heat-shock proteins (HSPs) interact with the immune system in a very complex manner. Many such HSPs exert powerful effects on the immune response, playing both stimulatory and regulatory roles. However, the influence of the HSPs on immunity appears to be positive or negative in nature – rarely neutral. Thus, the HSPs can act as dominant antigens and can comprise key components of antitumor vaccines. They can also function as powerful immunoregulatory agents and, as such, are employed to treat inflammatory diseases or to extend the lifespan of tissue transplants. Small modifications in the cellular milieu have been shown to flip the allegiances of HSPs from immunoregulatory agents toward a potent inflammatory alignment. These mutable properties of HSPs may be related to the ability of these proteins to interact with multiple receptors often with mutually confounding properties in immune cells. Therefore, understanding the complex immune properties of HSPs may help us to harness their potential in treatment of a range of conditions.

## Introduction

Molecular chaperones are proteins that permit the maturation and correct folding of most of the proteome (1, 2). As such, they are found in all cellular organisms and seem essential for cellular life. Protein folding seems to require chaperones from a number of different gene families that appear to function at various stages in a concerted folding cascade. These proteins belong to the small heat-shock protein (HSP) family including Hsp27 and the larger 70 kDa HSP family including Hsp70 as well as Hsp60, Hsp90, and Hsp110 families (3) (Table 1). We will discuss here, the mammalian immune responses to both prokaryotic (eubacterial) and eukaryotic HSPs under a range of contexts. The acronym HSP is derived from the early findings that some of these proteins are massively induced during proteotoxic stresses such as heat shock (4). Thus, the canonical functions of the HSP chaperones are in the folding of proteins during mRNA translation and in responding to protein unfolding crises in stressed cells (5).

**Table 1:** **Immune/inflammatory roles for extracellular chaperones**.

Chaperone	Pro/anti-inflammatory	Adaptive immunity?	Reference
Hsp27	Context	−	(5)
Hsp60	Context	−	(1, 6–8)
Hsp70	Context	+	(9–14)
Hsp90	Context	++	(14–18)
Hsp110	Pro	+++	(19–22)
Grp94	Pro	+	(23, 24)
Grp170	Pro	+++	(25–29)
Calreticulin	Pro	+	(23, 24)

However, HSPs also appear to possess functions outside the realm of protein folding, some of them acquired when they are released from cells to become extracellular HSPs (5, 6, 30, 31). HSPs have been observed in serum from human patients, pointing to their existence outside of cells, in living organisms (9). Among the first functions mooted for extracellular HSPs were in inflammation and immunity (23, 32). HSPs of each of the classes appeared to function in influencing the inflammatory and immunological balance in tissues (Table 1). The hypothesis of a pro-immune function for extracellular HSPs was derived primarily from studies utilizing molecular chaperone vaccines in cancer treatment (10, 23, 33). It was shown that HSPs from a number of chaperone families could be extracted from cancer cells while they were associated with a range of tumor peptide antigens (11, 33, 34). These HSP–peptide complexes could then be injected into hosts as anticancer vaccines, delivering a range of tumor-derived antigens to the immune system and promoting antitumor immunity (19–22, 25–29). HSPs were, by the proponents of this approach, conventionally, viewed as playing a dominant role as promoters of immunity (32). In addition, a number of studies showed them to be pro-inflammatory mediators, and extravagant claims were made for molecular chaperones as activators of multiple facets of immunity. However, other investigators have demonstrated powerful anti-inflammatory roles for HSPs that we will discuss more fully, later in this manuscript (12, 35, 36). In addition, the properties of extracellular HSPs have now expanded to include powerful roles in processes outside the immune response. For instance, secreted Hsp90 has been shown to mediate wound healing and tumor metastasis (36, 37). Thus, extracellular HSPs appear to have come of age as major intercellular signaling molecules in biology and medicine.

Some of the issues discussed here, particularly the role of HSPs in antigen presentation, have been mentioned in a previous review (38). Here, however, we focus mainly on the potentially confound pro- and anti-inflammatory roles of HSPs and discuss how these properties can be manipulated toward clinically useful outcomes in both treatment of autoimmune conditions and in the deployment of chaperone anticancer vaccines.

## Release of HSPs into the Extracellular Microenvironment

Structural considerations would tend to make one skeptical regarding the possibility of HSP secretion into the extracellular milieu. HSP family proteins lack an N-terminal hydrophobic signal sequence, characteristic of most secreted proteins, and thus, cannot be released from cells by the conventional secretion pathways. However, a number of non-canonical secretion pathways exist, many of which are employed by cytokines to gain access to the extracellular milieu. These eccentric mechanisms include release of the polypeptides *via* secretory lysosomes, a pathway utilized in the release of IL-1β from inflammatory cells (39). Hsp70 has been shown to be secreted from a number of cells in free form by a similar pathway, through a mechanism requiring the lysosomal pH gradient (31, 40). Indeed, Hsp70 is cosecreted from cells along with the lysosome resident protein LAMP1 (31). Hsp70 is also released from a range of other cells including tumor cells, reticulocytes, peripheral blood mononuclear cells, B cells, and dendritic cells in various types of lipid vesicles [reviewed by De Maio (41) and Vega et al. (42)]. These vesicles may include a variety of lipid-bounded structures, including ectosomes that are vesicles derived from the plasma membrane and that may contain cytosolic proteins as well as exosomes. Formation of exosomes is a complex process including the internalization of portions of the plasma membrane and subsequent release of exosomes containing a variety of previously intracellular proteins, including HSPs (43). The exosomal pathway is also utilized by some cells for IL-1β secretion (44). HSP-containing exosomes have a wide array of properties including both immunostimulatory and immunosuppressive functions, depending on the protein content of the exosome, cell of origin, and target cell (45–47).

Heat-shock proteins, therefore, can be secreted from a variety of cells in free form and in membrane-bounded particles. In addition, they can be released from cell undergoing necrotic death when membranes are disrupted, and the HSP can leak passively out of the cells (48). Hsp70 released in such a way has been shown to be strongly immunostimulatory.

## HSPs as Carriers of Tumor Antigens and Mediators of Immunity

### Adaptive Immunity

**Figure 1:**
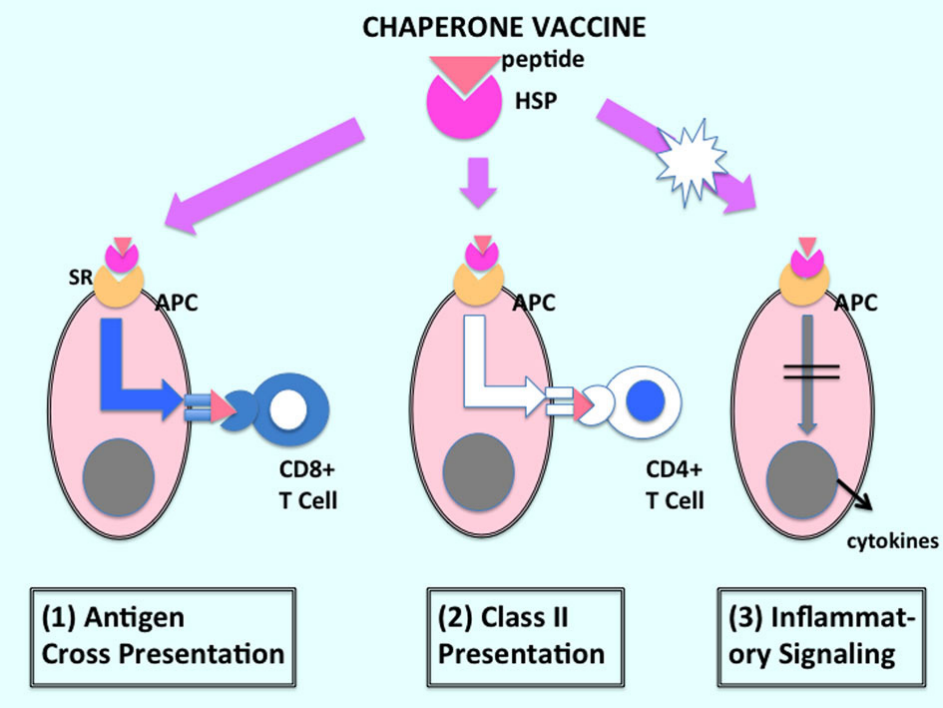
**Immune activation by HSP-based anticancer vaccines**. The HSP–peptide complexes that comprise the anticancer vaccine are shown to interact with APC after vaccination of host. The vaccines can efficiently (1) cause cross-presentation of tumor antigen and, thus, prime CD8^+^ T lymphocytes as well as activating CD4^+^ T cells through the (2) class II pathway. However, many investigations suggest that HSPs may not have major effects on (3) inflammatory signaling and may require combination with agents with adjuvant activity or inflammatory cell killing. Gray spheres indicate nuclei.

Molecular chaperones are unique immune modulators in that they can associate with a wide range of antigenic peptides and facilitate their delivery to antigen-presenting cells (APCs) (11, 13, 23, 33, 34). This property has proven to be desirable in the preparation of anticancer vaccines. Only a relatively small number of tumor antigens have been characterized, and we presume that this group represents a small minority of the real repertoire of unique cancer-derived antigens. Thus, chaperones, such as Hsp70, can be considered to “sample” the antigenic milieu of the malignant cell on encountering processed peptides *in vivo* and can be used to carry this sample into the APC during immunization (Figure 1). Such HSP-containing vaccines have proven to be highly effective in studies in experimental tumor systems in mice, in which they can lead to tumor regression associated with the generation of specific immunity (10, 13, 14, 20, 49–51). Issues in the preparation of the vaccines, which may influence the clinical effectiveness of vaccines, include the degree to which antigens can be retained by the chaperone and the affinity for the peptide during immunization and entry into APC (11, 14, 52).

Cross-presentation is a process by which extracellular antigens can gain access to the MHC class I pathway, a mechanism normally reserved for processing and presenting endogenous antigens (38). Efficient antigen cross-presentation is very important for vaccine effectiveness as MHC-I–peptide complexes permit recognition of cells bearing the complexes and killing by CD8^+^ cytotoxic T lymphocytes (53). Interestingly, Hsp90 appears to protect the integrity of internalized antigens associated with it, to trigger cross-presentation, and to carry antigens deep into the cell, penetrating the plasma membrane and endosomal membranes, and delivers chaperoned peptides to cytoplasmic proteasomes for processing (15, 16).

Although CD8^+^ T cells can be triggered by DC to recognize antigens after cross-presentation, in the absence of further signals, such T cells are unable to kill their targets. Other inputs are required for full activation (54, 55). The principal pathway used by APC for sampling external antigens is the MHC class II pathway. MHC class II molecules are found only on the surfaces of immune cells. The class II pathway involves the uptake of antigens by receptors on DC, processing of such antigens in the lysosomal compartment, transport of vesicles containing antigen–MHC-II complexes to the cell surface, and presentation to CD4^+^ T lymphocytes. Hsp90 is able to carry associated antigens into APCs and direct them into the class II pathway as well as facilitate cross-presentation. The choice of direction regarding entry of antigens into the class I/cross-presentation or class II pathways appears to usually depend upon the antigen-binding receptor that mediates triage between the two presentation systems (56, 57). However, Hsp90 appears neutral in this regard and increase penetration of associated peptides into either pathway (17, 18). One important activity governed by the MHC class II pathway is a process called dendritic cell licensing (54). In this mechanism, CD4^+^ T cells that recognize the antigen on a particular DC produce a reaction in the APC that permits it to activate CD8^+^ cells that interact with the same APC. Interaction of the T cell receptor on the CD4^+^ T cell triggers the expression of CD40 ligand (CD40L) that can bind the CD40 counter receptor on the DC and induce expression of inflammatory cytokines, such as TNFα and IL-12 as well as stimulatory coreceptors, like CD80 and CD86 (58, 59). These coreceptors cooperate with MHC class I in fully activating the CD8^+^ T cell through the T cell receptor. Thus, HSP–peptide complexes become internalized and trigger both the MHC class I and II pathways and may permit DC licensing to occur (17, 18). Our findings that HSPs can facilitate uptake of individual Ova antigens through the MHC-I and MHC-II pathways suggest the possibility of HSP–antigen complex could mediate DC licensing, although this has not yet been formally proven. Homing of CD8^+^ T cells toward licensed DC may involve surface chemokine receptor CCR5, a process strongly stimulated by chemokines, CCL3 and CCL4 (54). It has been shown in Lewis lung carcinoma cells, *in vivo*, that antitumor immunity was activated along with release of chemokines CCL2, CCL5, and CCL10, by a mechanism dependent on Hsp70 and TLR4 (60).

### Inflammation and Innate Immunity

On exposure to prokaryotic cells or cell products, a separate branch of immunity known as innate immunity is stimulated. In this process, molecules characteristic of individual pathogens including contrasting types of viruses and bacteria, known as pathogen-associated molecular patterns (PAMPs) herald the infection and prime the immune response (61). Then, PAMPs interact with specific receptors on macrophages or DC, known as pattern recognition receptors (PRR), and trigger innate immunity. Best known among the PRR are the toll-like receptors (TLR) that can couple binding of individual PAMPs to intracellular signaling pathways and gene expression programs (62, 63). Most notable among the mechanisms triggered by PRR occupation are the NKK and MAP-kinase pathways that influence inflammatory transcription through activation of factors, such as NFκB and IRF3 (64). This process can lead to synthesis of costimulatory molecules, such as CD80, and activating cytokines such as TNFα and IL-12 that synergize with MHC class I signaling in generation of active and long lived CTL (54). It is not clear to what extent HSPs derived from prokaryotes might function as PAMPs, although their extreme conservation across all cellular species would seem to argue against this. HSPs derived from mycobacteria are, however, recognized by the mammalian immune response and invoke powerful immunity to the extent that they have been described as superantigens (65). The mechanisms by which prokaryotic Hsp60 activates immunity are not clear but could involve PRR, such as TLRs, or other mechanisms.

It has also been shown that some molecules released from damaged and dying cells, such as uric acid and high mobility group box 1 protein (HMGM1), may trigger a form of sterile innate immunity, and such molecules are referred to as damage-associated molecular patterns (DAMPs), in order to suggest a functional similar to PAMPs (66, 67). Thus, DAMPs are thought to trigger innate immunity by binding to PRR and triggering inflammatory signaling cascades. Hsp70 was widely reported to function as a DAMP and to trigger innate immunity through the TLR2 and TLR4 pathways (32). Although this field has run into some controversy, the majority of findings in studies carried out *in vivo* over the past 15 years suggested that Hsp70, through interaction with TLR4, could potentially act as a DAMP (68). This area has been recently reviewed in depth (69). In addition to Hsp70, extracellular Hsp27 has recently been shown to cause both inflammatory and anti-inflammatory effects (5).

## Some Anti-Inflammatory and Immunoregulatory Effects of HSPs

Although prokaryotic HSPs can trigger a powerful immunodominant response in animals, most reports indicate that their effects are generally not pro-inflammatory, and the antibodies and T cells activated in the response had anti-inflammatory properties (69–71). Curiously, the epitopes that T cells respond to in mycobacterial Hsp60 were conserved with mammalian HSPs, and such cells recognized and responded to epitopes in the mammalian proteins. Prokaryotic Hsp60, therefore, did not seem to act as a PAMP (7). In addition, although HSPs were shown to interact with TLRs, such PRR often provoked anti-inflammatory signaling (8). For instance, Hsp60-derived peptides interacted with TLR2 on regulatory T cells (Tregs) leading to an immunosuppressive response. In addition, purified mycobacterial Hsp70 inhibited the maturation of DC (72, 73). Intracellular HSP levels were shown to increase in inflamed tissues and HSP-derived peptides expressed on the cell surface and appeared to activate Treg responses, thus mediating immunoregulatory functions (74). No studies, to date, have shown direct interactions between HSPs and TLRs, and in fact, attempts to show such binding have been negative (73, 75). The extracellular influences on TLR activity that have been reported may, therefore, be indirect and likely dependent on primary interactions of the HSP with other receptors on immune cells, such as the scavenger receptors (SR), followed by recruitment of TLR (64). The powerful immune effects of non-mammalian Hsp60 may also involve mechanisms independent of TLRs, and it has been suggested that the immune response may be genetically programmed to respond to such chaperones (76).

Interestingly, some of the studies applying HSP vaccines to cancer therapy indicated that, although there was significant activation of antitumor CTL by these agents, these were followed by a delayed Treg response. These findings suggest contrasting effects of the vehicle (HSP) and cargo (antigenic peptide) components of chaperone vaccines on immunity. These data might be interpreted as, suggesting that, while tumor antigens chaperoned by the HSPs trigger antitumor immunity, processed peptides from the HSP component of the vaccine led to a suppression of immunity. Using the chaperone vaccines at lower doses appeared to favor induction of CTL over the immunoregulatory response, perhaps by reducing the levels of HSP-derived peptides below a threshold (77, 78). It would seem that most chaperone vaccines, although efficiently triggering external tumor antigen presentation, do not deliver the inflammatory signal required to overcome antigenic tolerance (Figure 1). Such vaccines might be improved by use of adjuvants or pro-inflammatory forms of therapy, as discussed below.

## When HSPs Become Pro-Inflammatory Factors

One notable finding observed in multiple investigations of HSPs was that, even in studies where an inflammatory response to HSPs was not detected, the chaperones could strongly amplify responses to PAMPs, such as LPS (79). Mycobacterial Hsp65, a protein discussed in the last section as provoking generally immunomodulatory responses, when covalently fused to antigenic polypeptides produced a potent vaccine that generated effective CTL even in the absence of adjuvant (80). In addition, the combination of Hsp70 elevation in target tissues with therapies leading to necrotic cell killing led to a profound stimulation of inflammation and CTL killing that could lead to tumor rejection (81). This approach involved, after elevation of tissue Hsp70, targeting the normal tissues of origin with treatments that led to inflammatory modes of cell killing. This combined treatment resulted in the regression of distant, transplanted tumors (81). The findings observed in these studies were that, for instance, in prostatic tissue, cell killing by fusogenic viruses in the presence of elevated Hsp70 led to induction of the cytokines IL-6 and TGF-β, resulting in generation of highly inflammatory IL-17 and tumor rejection by antigen-specific CTL (82). This effect seemed to depend on generation of IL-6 by the combination of high tissue levels of Hsp70 and inflammatory death. However, in similar studies on pancreatic tissues, combination of Hsp70 and lytic virus failed to generate IL-6 and led to generation of a Treg response and continued growth of pancreatic carcinoma (82). Thus, the balance between immunoregulatory and immunogenic responses of Hsp70 appears to be poised on a knife-edge, influenced by the tissue type and mode of cell killing.

**Figure 2:**
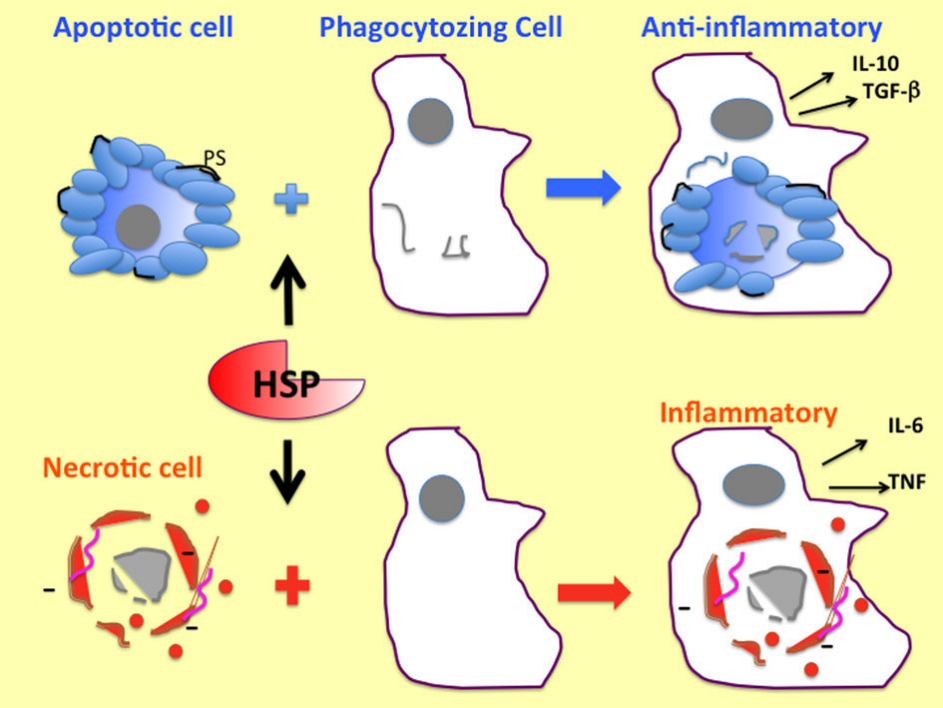
**Contrasting effects of cell corpses resulting from apoptotic and necrotic cell killing on immunity**. Apoptotic cells, depicted here with surface blebs, externalize the phospholipid phosphatidylserine to the outer leaflet of the plasma membrane leading to anti-inflammatory signaling and suppression of immunity. Necrotic cells depicted here with compromised membranes release DAMPS, such as HMGB1 (small orange spheres), and acquire net negative charge. Nucleic acids (pink lines) become located to the cell surface. Necrotic cells often trigger release of inflammatory cytokines, such as IL-6 and TNFα. HSPs, such as Hsp70, trigger the phagocytosis of dead mammalian cells as shown here, although the mechanisms involved in such stimulation are currently not clear. Gray spheres indicate nuclei.

It is well known that the mode of cell death has a powerful influence on inflammation and immunity in interacting APC (83). For instance, when cells die by an apoptotic mechanism, their intracellular contents remain enveloped by an external membrane and, thus, are not released into the environment to trigger inflammation (Figure 2). In addition to this, many apoptotic cells expose “eat me” signals, such as the phospholipid phosphatidylserine on the surface, triggering engulfment by macrophages and leading to immunosuppression (84, 85). Additional anti-inflammatory signals emanating from apoptotic cells may include the release of AMP from the apoptotic cell (86). In necrotic cell death, cell membranes become permeabilized, the intracellular milieu becomes externalized, and DAMPs, such as HMGB1, urate, and nucleic acids, released in this way become accessible to detection by neighboring macrophages or DC (85). It should be noted that the response of phagocytic cells to apoptotic bodies is complex and depends on the nature of the dead or dying cells and the surface densities of “eat me” or “don’t eat me” signaling molecules that are cell specific. In addition, in late apoptotic cells that have failed to be phagocytosed at an early stage, membranes become permeabilized. Therefore, such late apoptotic cells acquire some of the properties of necrotic cells, permitting release of DAMPs and switching the effects of the cell corpses on engulfing phagocytes toward a more immunogenic influence (85, 87).

**Figure 3:**
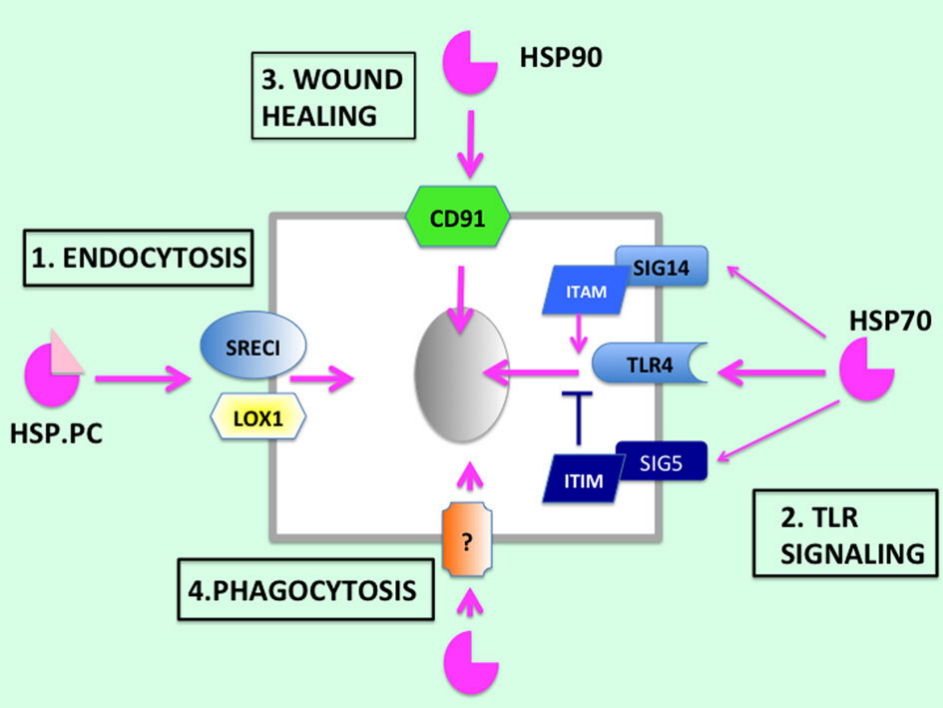
**HSP receptors**. HSP receptors include SREC-I and LOX-1 that mediate (1) endocytosis and, thus, presentation of antigens to APC. Hsp70 can also (2) trigger signaling through the TLR4 pathway in a range of cells. (It is not clear whether HSPs can interact directly with TLRs or whether the primary interactions are through other receptors.) Effects of Hsp70 on TLR4 signaling can be modulated by binding to either Siglec-14 that activates TLR4 signaling or other Siglec family members, such as Siglec-5, that activate the pathway. Hsp90 can also (3) activate wound healing responses, which may play key roles in inflammation, through binding to CD91. Hsp70 triggers (4) phagocytosis, a property that may be important in its immune functions, through currently unknown mechanisms. Gray ovals indicate nuclei.

It is not clear in which way extracellular HSPs might synergize with the extruded contents of necrotic cells in activating immunity, although increased capacity for engulfment by immune cells which is triggered by Hsp70 might amplify inflammatory functions (88–90). The earlier studies of Todryk et al., in fact, indicated that extracellular Hsp70 decrease DC maturation and increase capacity for engulfment of antigenic materials by the immature APC (91). Phagocytosis is a complex process and involves the formation of a broad synapse type structure between the engulfing cell and the mammalian cell body of prokaryotic cells. Multiple receptors appear to make contact between the cells. Interestingly, in *C. elegans* this process has been shown to require the surface receptor CED-1 (92). Both, mammalian scavenger receptor associated with endothelial cells (SREC-I) and cluster of differentiation 91 (CD91) possess sequence similarities to this protein, suggesting some involvement in phagocytosis and a potential mechanism for HSPs in this process (93). In addition, necrotic cells can supply signals to augment the immune effects of HSPs through release of HMGB1, a ligand for TLR4, and externalized nucleic acids that might interact with TLR3 or TLR9 and trigger inflammatory signaling (85) (Figures 2 and 3).

## Potential Mechanisms – Cell Surface Receptors and Cell Signaling Pathways

Most evidence suggests that the biological effects of extracellular HSPs are mediated through cell surface receptors (24, 94) (Figure 3). Thus, some of the clues to the properties of extracellular HSPs may lie within the interactions of these chaperones with these receptors and the unleashing of their embedded signaling cascades. The first surface receptor reported to bind to HSPs was CD91 also known as Low density lipoprotein receptor-related protein 1 (LRP1), the alpha-2-macroglobulin receptor (A2MR), and apolipoprotein E receptor (APOER) (24). This high molecular weight receptor was shown originally to bind to LDL and, as its possession of multiple names suggests, a wide range of (over 30) other extracellular structures (94). CD91 is expressed most abundantly in vascular smooth muscle cells and in hepatocytes. There has been some skepticism regarding its expression in APC, such as DC, and, therefore, its potential role in immune responses involving the HSPs (95). However, there seems little doubt that CD91/LRP1 is a *bona fide* HSP receptor, and this protein appears to play key roles in, for instance, responses to Hsp90α in the wound healing response and in tumor metastasis (36, 37).

It was next shown that Hsp70 could associate with lectin-type oxidized LDL receptor (LOX-1), a receptor found on the surfaces of human DC and that such binding could mediate cross-presentation of peptide cargo associated with this HSP, leading to CD8^+^ lymphocyte-mediated immunity (75). LOX-1 is the product of the oxidized low-density lipoprotein receptor 1 (*ORL1*) gene expressed most abundantly in vascular endothelial cells, macrophages, and DC (96). Although LOX-1 belongs to the C-type lectin receptor family, it also clusters with the SR, a group of proteins that have, in common, the ability to bind to covalently modified LDL molecules, such as oxidized LDL and acetylated LDL (97). Furthermore, exploration of the SR family indicated significant interaction of Hsp70 with at least two other members, including SREC-I, encoded by the *SCARF1* gene and Stabilin-1/FEEL-1 (encoded by the *STAB1* gene) (73, 98). SREC-I/*SCARF1* was able to bind to Hsp70 and Hsp90 in DC and mediated cross-presentation of associated tumor antigens, leading to activated CTL (13, 17). Murine bone marrow derived DC appeared to utilize both LOX-1 and SREC-I/*SCARF1* in interacting with Hsp90–peptide complexes (17, 75). In addition, SREC-I was also shown to mediate the uptake of antigens chaperoned by Hsp90 into the Class II pathway and stimulate activation of CD4^+^ T lymphocytes (18). The SR are regarded as receptors that respond to cellular debris including cell bodies, the remains of endogenous proteins spilled into the extracellular milieu, and the residue from invading pathogens. As such, the SR might be regarded as good choices for receptors with which APC might sample the extracellular environment (93). Stabilin-1 appears to be only sparsely expressed on the surface of DC and appears to function mostly in intracellular trafficking (99). Thus, there appeared little doubt that that the SR could bind to HSP-PC and mediate presentation of peptide cargo (17, 18). However, the nature of the receptors that might respond to HSPs and modify the inflammatory response still required investigation.

Many reports suggested that TLRs, particularly TLR2 and TLR4, might mediate inflammatory responses to HSPs in an up or down manner (32, 76). However, as mentioned above, attempts to demonstrate direct binding of HSPs to TLRs have not been generally successful (73, 75). Thus, HSP interaction with TLRs is likely to be an indirect one. It was shown, recently, that SREC-I/*SCARF1* could interact with TLR4 on the surface of mouse macrophages (64). Interestingly, both LPS and Hsp90 could mediate this complex interaction and lead to the sequestration of TLR4 in lipid raft domains (64). In the case of LPS, encasement of SREC-I–TLR4 complexes within lipid rafts was required for activation of signaling through the NFκB and MAP-kinase cascades, resulting in inflammatory cytokine secretion. Significantly, exposure to Hsp90, although leading to SREC-I–TLR4 colocalization, failed to trigger the inflammatory cascade through this mechanism. Alternative pathways of inflammatory suppression by Hsp90 may, thus, be involved (64).

Recent studies have also suggested that another receptor family, sialic acid-binding immunoglobulin-like lectins (Siglecs) may participate in inflammatory responses resulting from binding to HSPs (100). These receptors have been shown to bind to conjugated sugar residues in the cell coats of adjacent cells and generally suppress inflammation. Suppression of inflammation by these receptors involves immunoreceptor tyrosine-based inhibitory motif (ITIM) sequences in the intracellular domain that recruit anti-inflammatory proteins after ligand-triggered tyrosine phosphorylation (100). Upon activation, Siglec receptors have been shown to directly associate with TLRs and inhibit TLR-mediated activation of inflammatory signaling cascades, such as the NFκB pathway (101). Interestingly, recently, it was shown that two human Siglecs, Siglec-5 and Siglec-14 each, bind to Hsp70 (102). What was most intriguing about these interactions was that, while Siglec-5 contained the expected ITIM domain and repressed anti-inflammatory signaling through quenching NFκB, Siglec-14 appeared to have acquired a domain that binds proteins, such as DAP12, containing an intracellular immunoreceptor tyrosine-based activating motif (ITAM) that stimulated release of inflammatory cytokines (102, 103). Differential expression of members of the Siglec family might, thus, either amplify or suppress the HSP regulated activities of cell surface TLR and inflammatory signaling through mediation of the NFκB pathway. A further property of extracellular HSPs mentioned above, that may play a key role in their immune functions, is their ability to trigger phagocytosis. However, the mechanisms and receptors involved in this process have not been well characterized and further experiments will be required to understand the mechanisms involved (Figure 3).

Thus, responses to extracellular HSPs appear to involve the cooperative or confounding outputs of a range of cell surface receptors that together may determine their influence on immune reactions within tissues. To date, however, we have not been able to identify a dedicated high-affinity HSP receptor. Dedicated receptors, such as the insulin receptor, are often able to pick up tiny concentrations of ligands, often with affinities in the range of 10^−9^–10^−10^M, with exquisite selectivity. For HSPs, we have identified, so far, only “hand me down” receptors that also bind a large range of other ligands with moderate affinity. It remains to be determined if dedicated HSP receptors will be found in immune cells of mammalian species.

## Conclusion

The intracellular roles of HSPs in protein folding have been conserved since the dawn of cellular life (3). However, the HSPs also appear to have acquired key roles in the immune systems of animals early during evolution, and these roles are preserved in modern animal species (104). Such HSPs can capture intracellular antigens and present them to APC, mediating the cross-priming of recipient cells. In addition, HSPs, when processed and presented on the APC cell surface, can activate Treg cells and inhibit immunity and inflammation. Their effects on the immune system are, thus, bivalent. In the presence of PAMPs or tissues undergoing necrosis, Hsp70, in particular, becomes a strong inflammatory agent. The precise nature of the responses elicited by extracellular HSPs may, therefore, depend upon the particular tissue milieu within which they are released and the identities of the receptors on the surfaces of immune cells that encounter them.

## Outstanding Questions and Potential Advances

(1) Many of the immune-active HSPs are members of different gene families, with only a modicum of structural similarity (Table 1). However, each chaperone appears to be recognized by similar families of receptors, including SR and the LDL receptors, despite their sequence dissimilarity (94). Understanding the structural basis for extracellular HSP recognition by these and potentially new classes of receptors would be an advance in determining the roles of the chaperones in immunity and predicting novel recognition structures. However, this has been an open question for a number of years now and research is ongoing.
(2) It is becoming apparent that members of individual HSP gene families have distinct properties. For instance, only Hsp90α is secreted from cells and the other major isoform, Hsp90β, is mostly retained within the cell (36). It will be enlightening to learn whether such specificity in extracellular function holds for other HSP families.
(3) We have, in recent years, acquired some understanding of how these “leaderless” proteins gain access to the extracellular microenvironment in intact cells (see Release of HSPs into the Extracellular Microenvironment), although it will be invaluable to gain a more concerted understanding of mechanisms of HSP secretion.
(4) We will continue the search for HSP receptors in immune cells, their expression patterns and their connection to inflammatory and anti-inflammatory responses to both endogenous HSPs and prokaryotic paralogs (94). It is likely that a major key to understanding how chaperones trigger immune responses and the direction of such responses depends on understanding the combinatorial effects of the multiple receptor families.
(5) It seems clear that many HSPs, although capable of efficient transport of antigenic peptides within chaperone vaccines, do not on their own deliver a second signal for APC maturation, thus reducing their effectiveness as stand-alone agents. In order to break immune tolerance to tumor antigens, chaperone vaccines might be best deployed in cancer therapy in combination with PAMPs, such as double stranded RNA or unmethylated CpG motifs or checkpoint inhibitors such as anti-CTLA4 (105, 106). Alternatively, vaccines could be used with agents, such as ionizing radiation, that can cause necrotic killing and subsequent inflammatory effects in tumors (including release of intracellular DAMPs) thus leading to immune rejection of the cancers (10).

## Author Contributions

SC, JG, and AM contributed in writing the text of the review.

## Conflict of Interest Statement

The authors declare that the research was conducted in the absence of any commercial or financial relationships that could be construed as a potential conflict of interest.
